# Hospital Sulphate of Quinine

**Published:** 1853-03

**Authors:** 


*Hospital Sulphate of Quinine.*—Mr. Edward Herring has introduced a preparation under this name, consisting of disulphate of quinine only partially purified. In its medicinal properties it is said to differ but little from the ordinary disulphate. It has a brownish color, and is of course not admissible as a substitute for disulphate of quinine in general dispensing, but it has been tried in hospitals and dispensaries, and by some medical men who dispense their own medicines. The preparation is recommended on account of its economy. The final purification and decolorization of the salt being attended with some expense, the manufacturer is enabled to offer it in a partially purified state at a considerable reduction from the price at which it can be sold when purified in the usual way. The amount of impurity must be ascertained before its real value can be estimated. It may be a question whether the recognition of a preparation so imperfectly purified might not open the door to some abuse.—*Ibid.*

